# Atomic structure and crystallography of joints in SnO_2_ nanowire networks

**DOI:** 10.1007/s42649-019-0003-7

**Published:** 2019-04-29

**Authors:** Viktor Hrkac, Niklas Wolff, Viola Duppel, Ingo Paulowicz, Rainer Adelung, Yogendra Kumar Mishra, Lorenz Kienle

**Affiliations:** 1 Synthesis and Real Structure, Institute for Materials Science, Kiel University, Kaiserstr. 2, 24143 Kiel, Germany; 2 Nanochemistry, Max Planck Institute for Solid State Research, Heisenbergstr. 1, 70569 Stuttgart, Germany; 3 Phi-Stone AG, Kaiser Str. 2, 24143 Kiel, Germany; 4 Functional Nanomaterials, Institute for Materials Science, University of Kiel, Kaiser Str. 2, 24143 Kiel, Germany

**Keywords:** Electron microscopy, Tin dioxide network, Flame transport synthesis, Precession electron diffraction, Atomic interface

## Abstract

Joints of three-dimensional (3D) rutile-type (r) tin dioxide (SnO_2_) nanowire networks, produced by the flame transport synthesis (FTS), are formed by coherent twin boundaries at (101)^r^ serving for the interpenetration of the nanowires. Transmission electron microscopy (TEM) methods, i.e. high resolution and (precession) electron diffraction (PED), were utilized to collect information of the atomic interface structure along the edge-on zone axes [010]^r^, [111]^r^ and superposition directions [001]^r^, [101]^r^. A model of the twin boundary is generated by a supercell approach, serving as base for simulations of all given real and reciprocal space data as for the elaboration of three-dimensional, i.e. relrod and higher order Laue zones (HOLZ), contributions to the intensity distribution of PED patterns. Confirmed by the comparison of simulated and experimental findings, details of the structural distortion at the twin boundary can be demonstrated.

## Introduction

The capability of developing functional (nano-)materials for complex or high-tech applications originates in the deliberate control of innovative and sophisticated manufacturing processes (Davis 2002; Dick et al. 2004; Mathur et al. 2005). In the field of stretchable and porous ceramics, the flame transport synthesis (FTS) emerged as a unique method for a rapid production of macroscopic amounts of oxide semiconductor or ceramic materials with tunable properties (Mishra et al. 2013). With variation of process parameters and utilization of various metal oxides (e.g. ZnO, Fe_2_O_3_, Al_2_O_3_, TiO_2_) a fabrication of several three dimensionally interconnected network systems is realized. The latter can be categorized into different synthesis classes, forming all by the combination of quasi one-dimensional (Q1D) nano−/microstructure building blocks, as described elsewhere (Xia et al. 2003; Zhang et al. 2003). In this manner, flexible macroscopic materials with the advantageous properties of ceramics are formed, enabling the application in demanding fields such as light-weight space technologies, high-temperature flexible sensors or stretchable implants (Rice 1998; Klawitter and Hulbert 1971; Sousa et al. 2003).

The junctions of the building blocks are based on structural defects at the nanoscale. Thus, the mere presence, concentration and type of these defects define the morphology, the physical properties, e.g. piezoelectricity, of such a network and subsequently the efficiency/ performance of potential devices (Molarius et al. 2004). In other words, the enhancement or intended manipulation progression of certain properties is driven by a complete understanding of the real structure-to-property relation. As immanent task the investigation of these defects with adequate measuring equipment becomes compulsory: Conventional and advanced transmission electron microscopy (TEM) enables an analytical approach of studying such defects and deducing required structure models (Hrkac et al. 2015)^.^

In this work, detailed TEM investigations are reported for the junction forming structural defects, i.e. twin boundaries, of FTS synthesized flexible 3D networks based on tin oxide (SnO_2_). Property and basic structural investigations are provided in previous works elsewhere (Paulowicz et al. 2015). It must be emphasized, that the understanding of structural defect phenomena observed in TEM lies in the possibility of their computational simulation and, thus, in their quantitative description. The main challenge is the combination of comparatively uncomplicated simulations of periodic structures with those of non-periodic objects, e.g. twin boundary, planar defect, antiphase boundary. Although models were already described for SnO_2_ defect structures, a substantial improvement can still be achieved, especially considering the simulation of the high-resolution contrast by applying a supercell approach. This approach (Deiseroth et al. 2004; Kienle and Simon 2002) provides such upsides and is used to transform common unit cells into elaborated defect models using exclusively basic crystallographic principles. Further a step-by-step guide is provided to generate a complete 3D model of the SnO_2_ twin boundary, which is verified by cross-sectional and superposition TEM data of the defect structure.

## Material and methods

Details of the synthesis process can be found elsewhere (Mishra et al. 2013). Scanning electron microscopy (SEM) studies were conducted with a Carl Zeiss microscope (10 kV, 10 μA). High resolution transmission electron microscopy (HRTEM) has been realized with a Philips CM 30 ST microscope (LaB_6_ cathode, 300 kV, C_S_ = 1.15 mm). Sample preparation for TEM was carried out by a grinding method and subsequent placement of the specimen on a lacey carbon/copper grid. This grid was fixed in a side-entry, double-tilt holder with a maximum tilting of ±25° in two directions. A spinning star device enabled precession electron diffraction (PED) (Schürmann et al. 2011). Simulations of HRTEM micrographs and PED patterns have been calculated using the JEMS program package (Stadelmann 1987) and in particular a multi-slice formalism (JEMS preferences: spread of defocus: 70 Å, illumination semi-angle: 1.2 mrad). For the evaluation of ED patterns and HRTEM micrographs (including Fourier filtering) the program Digital Micrograph 3.6.1 (Gatan) was used. A contrast enhancement of the micrographs was performed with a HRTEM filter plug-in for DM (Kilaas 1998). Chemical analyses by energy-dispersive X-ray spectroscopy (EDS) were performed with a Si/Li detector (Noran, Vantage System).

### Supercell approach

**Fig. 1:**
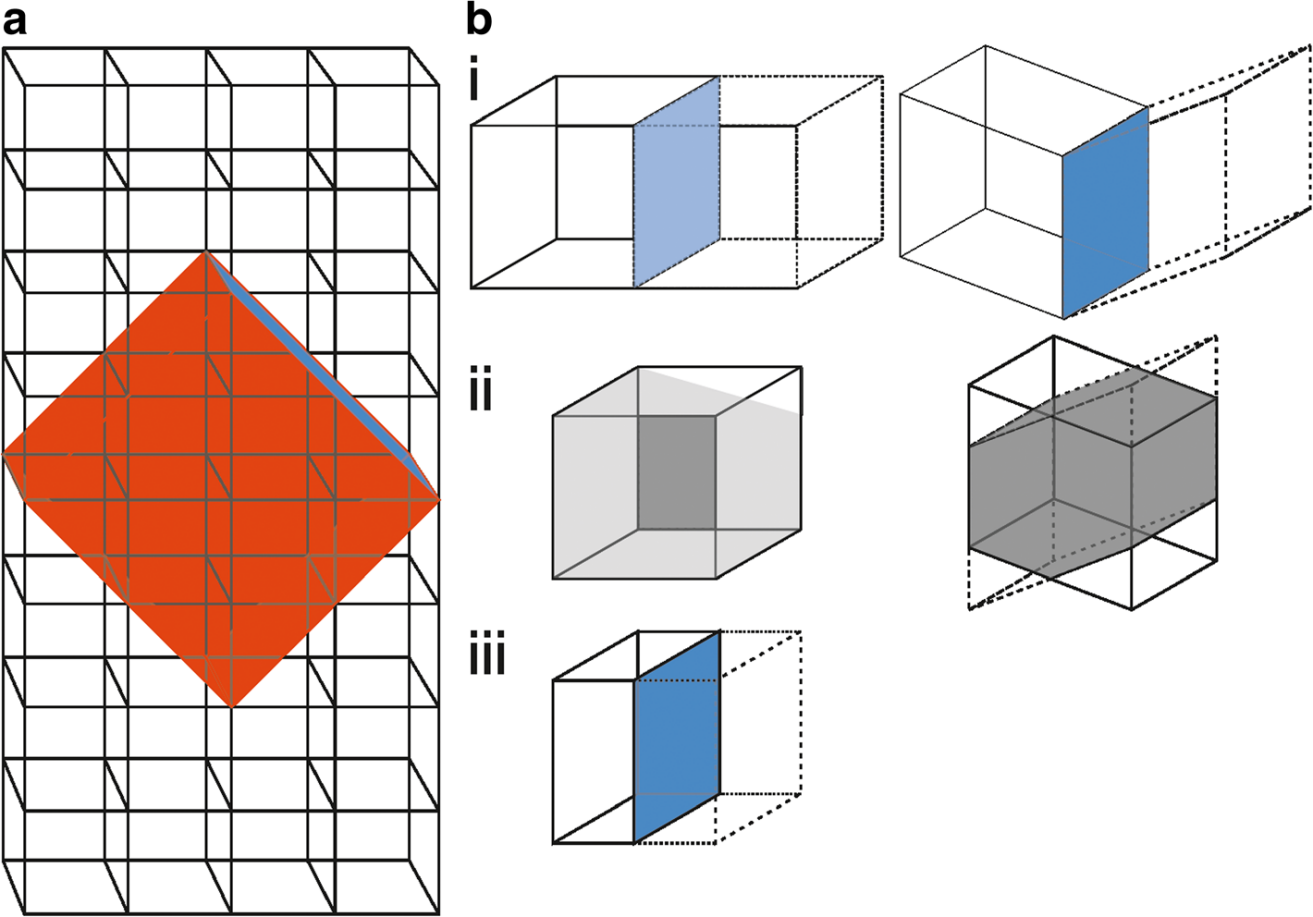
Supercell approach: **a** Stylized representation of periodic crystal (black bordered cubes as unit cells). The supercell (red cuboid) is extracted from the periodic crystal with one facet (blue plane) as embedded defect within the crystal. **b** Comparison: supercells with a defect plane (blue) fulfilling (left) and violating (right) the coincidence condition for the superposition and separation steps: i) Inversion/ mirroring of the supercell along the defect plane. ii) Superposition of the supercell with its inversed duplicate. iii) Separated SPS including the defect plane as domain boundary

A planar defect of the crystal structure is converted into a pseudo periodic feature by embedding into a suitable supercell. The entire supercell approach subdivides into three general steps:

The ideal structure from the material of choice must be transformed into a triclinic (*P*1) structure considering the defect type and its orientation, cf. Fig. 1a. As a consequence of the symmetry reduction, all symmetry restrictions according to the ideal structure are eliminated with a simultaneous preservation of all these symmetry elements as pseudo-symmetry elements. Note, optimized metrics of the supercell, i.e. all angles of the generated cell are restricted to 90°, may be preferred or are essential for specific defect types. Otherwise the description of both domains separated at the defect in one common unit cell is inhibited. Any deviation from this coincidence condition can result in severe deviations between final simulations and experimental observation (Hrkac et al. 2013). The mathematical formalism of an unit-cell transformation into an unconventional description is given in (Hahn 2002):

$$ \left({a}^{\hbox{'}},{b}^{\hbox{'}},{c}^{\hbox{'}}\right)=\left(a,b,c\right)P, and\left(\begin{array}{c}{u}^{\hbox{'}}\\ {}{v}^{\hbox{'}}\\ {}{w}^{\hbox{'}}\end{array}\right)=Q\left(\begin{array}{c}u\\ {}v\\ {}w\end{array}\right)\ \mathrm{with}\kern0.5em Q={P}^{\hbox{-} 1} $$

(*a*, *b*, *c*) represent the basis vector of the direct space, *u*, *v*, *w* are indices of a direction in direct space, (^'^) as mark of the parameters for the *P*1 cell: *P* and *Q* as (3 × 3) square matrices, linear parts of an affine transformation.

The characteristic symmetry element of the defect type defines the number and the construction manner of modified supercells, see the example in Fig. 1b: The blue side surface in the sketch represents a twin interface (mirror plane) separating the original supercell with its mirrored counterpart (Fig. 1b-i, left). By superimposing and merging these cells into one, so-called superposition structure (SPS) can be constructed (Fig. 1b-ii, left). Violating the 90° (coincidence) restriction will lead to an artifact-containing SPS, cf. Fig. 1b-i/ -ii, right.

An isolation or segregation of individual domains within the SPS is executed which creates a new (separation-)cell with the domain boundary as center part, see Fig. 1b-iii.

## Results and discussion

**Fig. 2:**
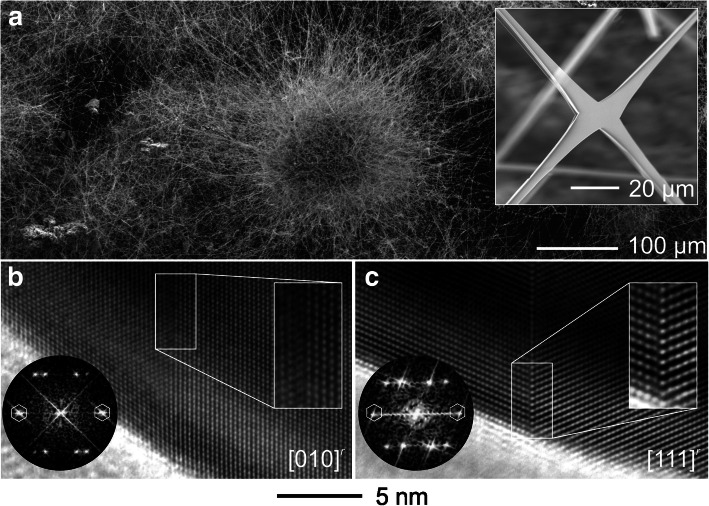
Three-dimensional network composed of interconnected SnO_2_ micro- and nanostructures: Scanning electron micrographs of **a** an overview network and a representative junction (inset). High resolution transmission electron micrographs of the SnO_2_ twin interface with the (101)^r^ coherent twin boundary along the **b** [010]^r^ and **c** [111]^r^ zone axes. Circular insets: respective Fast Fourier Transformation patterns with the 101 intensities marked. Rectangular insets: emphasized views on the (101)^r^ twin boundary

The fibrous morphology of the 3D SnO_2_ network is illustrated in Fig. 2a. This FTS network is constructed by nano−/ microwires and nanobelts interconnecting via junctions as exemplarily demonstrated in the inset of Fig. 2a. A detailed structural characterization of such features was carried out by TEM: All obtained data confirm a rutile type structure (index label: *r*) with the tetragonal space group *P*4_2_/*mnm*. Chemical impurities within the investigated areas can be excluded via accompanied EDS analyses which exhibited exclusively a composition of SnO_2_. The appearance of twinning defects was a frequently observed feature, as exemplary presented in HRTEM micrographs of Fig. 2b, c. In both cases, the analysis of fast Fourier transform (FFT) pattern identifies the common twin plane to be {101}^r^ with a rotation angle between the mirrored domain individuals of 68.5°. Note, in all investigated cases only this twin type was observed. Following previous studies (Zheng et al. 1996) the observed twin can be classified as growth twin consisting of a coherent twin boundary (CTB). Additionally, a tendency of interpenetration twinning was reported for the CTB explaining the complex macroscopic morphology of single crystals and the entire networks.

### Model

#### Transformation of the ideal SnO_2_ structure

**Fig. 3:**
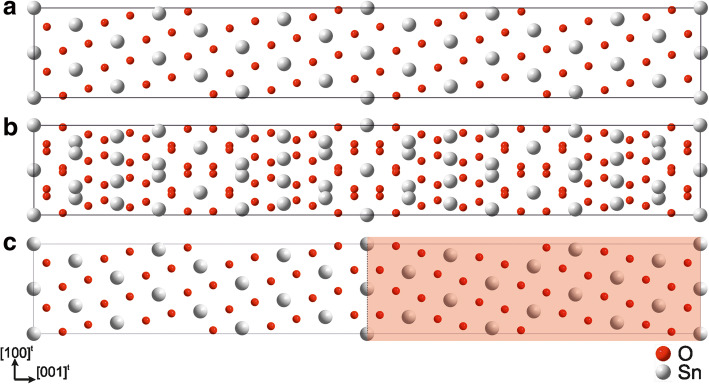
Supercell approach for the coherent twin boundary (101)^r^/[010]^r^ of the SnO_2_ rutile type structure: **a** Supercell, **b** SPS and **c** basic separation model. See text for details

The tetragonal rutile-type structure (space group: *P*4_2_/*mnm*) is transformed to a rectangular *P*1 supercell by applying the matrices (Fig. 3a):

$$ P=\left(\begin{array}{ccc}1& 0& 1\\ {}0& 1& 0\\ {}-5& 0& 11\end{array}\right),Q=\left(\begin{array}{ccc}11/16& 0& 5/16\\ {}0& 1& 0\\ {}-1/16& 0& 1/16\end{array}\right) $$

The new cell parameters are a’ = 0.5706 nm, b’ = 0.4735 nm, c’ = 4.227 nm and α’ =90°, β’ = 90.14°, γ’ = 90°. Note, for the upcoming steps and simulations β’ is set to 90° in order to fulfill the coincidence condition. This approximation is legitimate as the slight deviation results in a negligible error. Further, all transformed indices are labeled with *t.* In notation of the supercell, the directions [010]^r^, [111]^r^, [001]^r^ transform to [010]^t^, [110]^t^ and [501]^t^, respectively.

#### Creation of the SPS

A second supercell is created by mirroring its atomic positions with respect to the twin boundary. With a subsequent superimposition of both supercells a SPS is obtained, cf. Fig. 3b.

#### Separation and shift

**Fig. 4:**
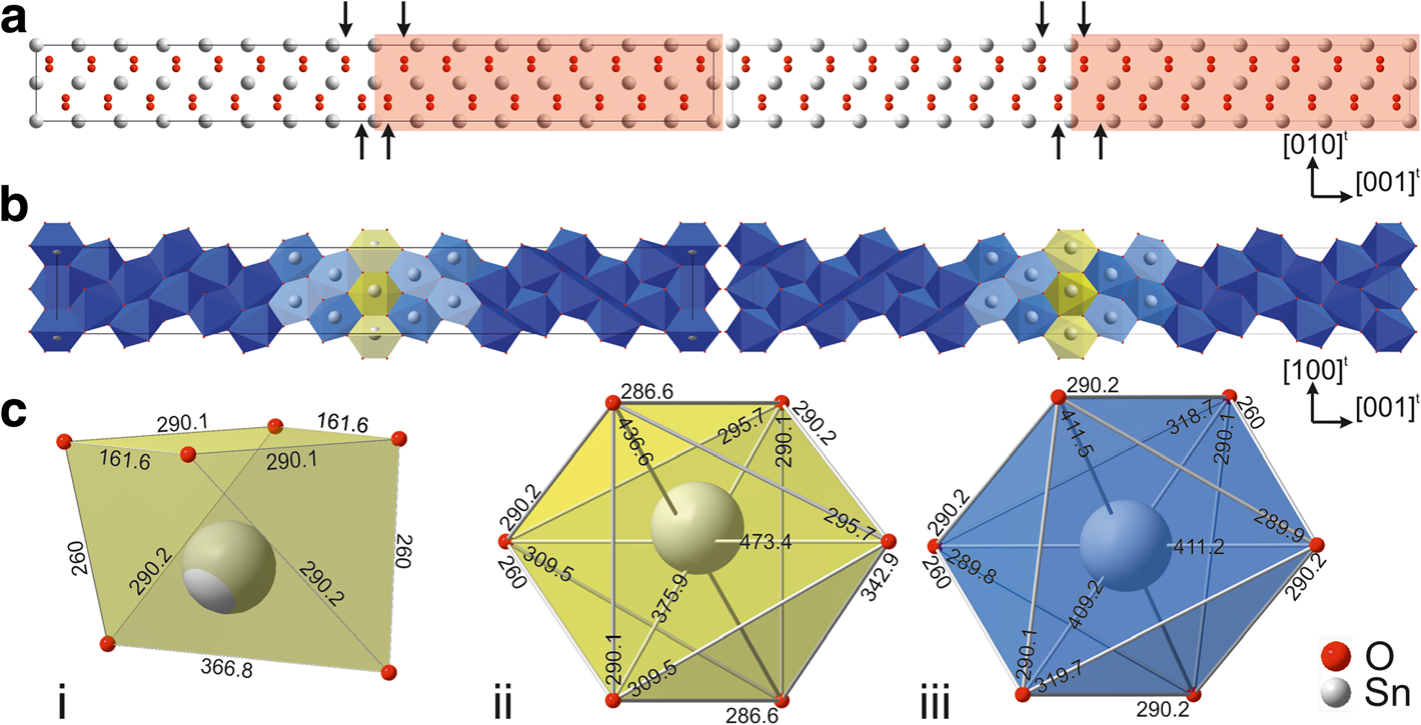
Basic (left) vs. improved (right) defect model along the **a** [100]^t^ and **b** [010]^t^ direction. **c** Excerpted SnO_6_ octahedrons: coherent twin boundary from i) basic vs. ii) improved models and iii) bulk octahedron, values are given in picometer. See text for details

The number of atoms of the SPS has been reduced by deliberately eliminating one supercell individual from each half cell, see plain and red marked regions from Fig. 3c. Additionally, the redundant duplicates of the central atoms (fine dotted line) were removed. Thus, this basic separation model consists of two single domains mirrored at an incorporated twin boundary. The atomic distances at the interface are modified to match up with the corresponding distances within the bulk by applying a shift vector ($\frac{1}{2}$ [010]^t^) on one single domain of the defect model, as demonstrated by the arrows in Fig. 4a. A polyhedral representation of the basic and improved defect model (Fig. 4b and c-i, c-ii) emphasizes the necessity of the vector: Only the improved model provides meaningful atomic distances in the vicinity of the central Sn atoms, cf. yellow marked octahedrons in Fig. 4b, c.

As the twin boundary became an incorporated feature of the supercell, simulations of multiple twinning are also enabled: A combination of slightly modified cells from step 1 and 3 can be used to tailor adequate three-dimensional (multi-)defect models.

Moreover, the atomic coordinates of the derived supercells and corresponding transformation matrices can be adapted for other rutile-type structures which contain the same {101}^r^ twin defect, e.g. considering different c/a ratios.

The improved model enables further the focused accentuation of the different SnO_6_-octahedrons, namely the CTB and defect-free bulk types (see Fig. 4c), and the determination of the degree of distortion for these octahedrons at the CTB. The atomic distances differ up to ca. 15%. Note, ab-initio calculations and experimental observations with aberration corrected TEM could provide enhanced information of the structural nature at the CTB, particularly about oxygen atom positions, and support probable modification of the defect model.

**Fig. 5:**
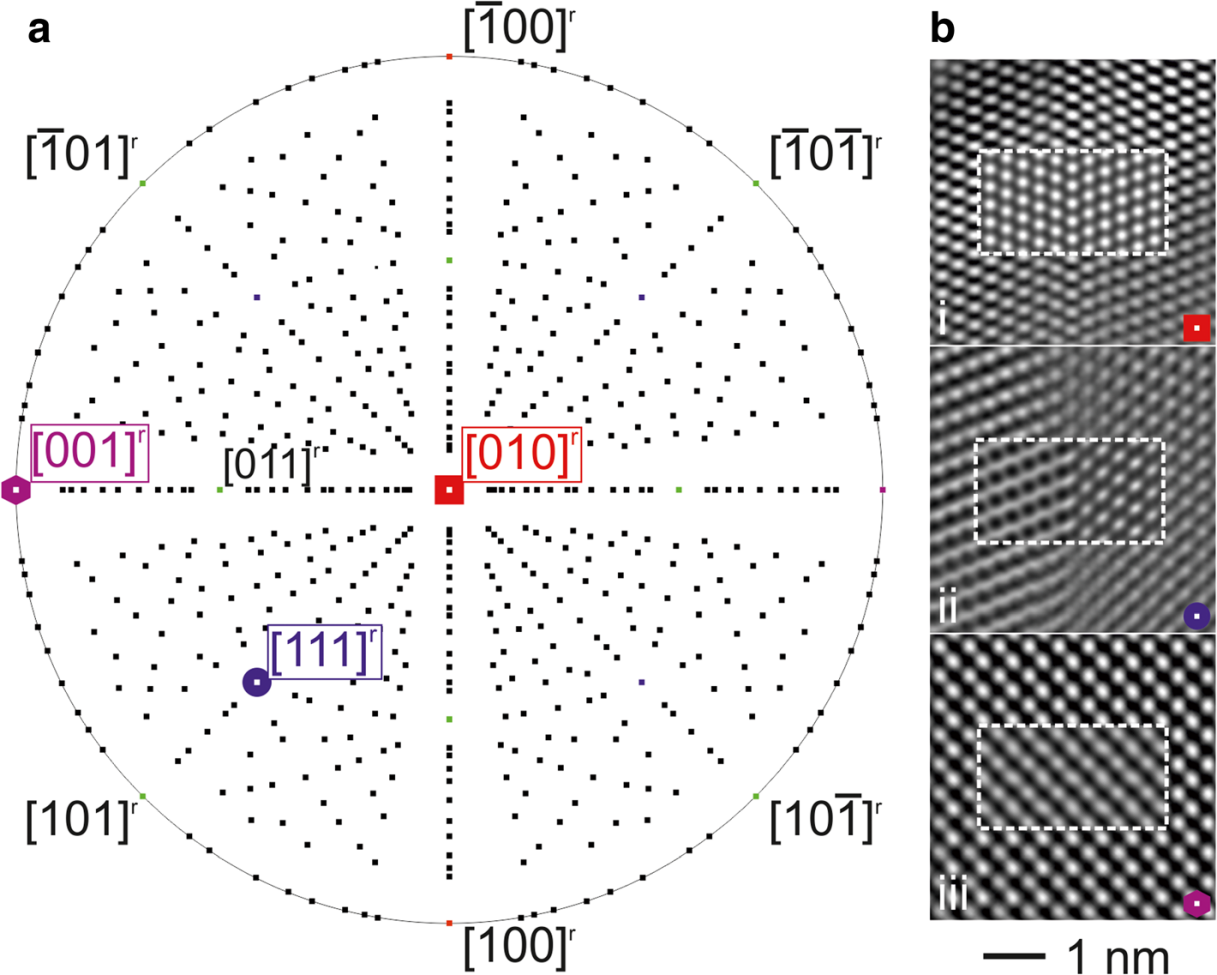
**a** Stereographic projection of the rutile type SnO_2_ with emphasis on the experimentally studied zone axes. **b** High resolution micrographs with simulations based on the defect model as inset along -i) [010]^r^, −ii) [111]^r^ and -iii) [001]^r^. See text for details

An in-depth analysis of the twinned rutile type structure is carried out by performing a multiple zone axis study; see highlighted zone axes in the stereographic projection of Fig. 5a.

### Tilting experiments I – real structure

**Fig. 6:**
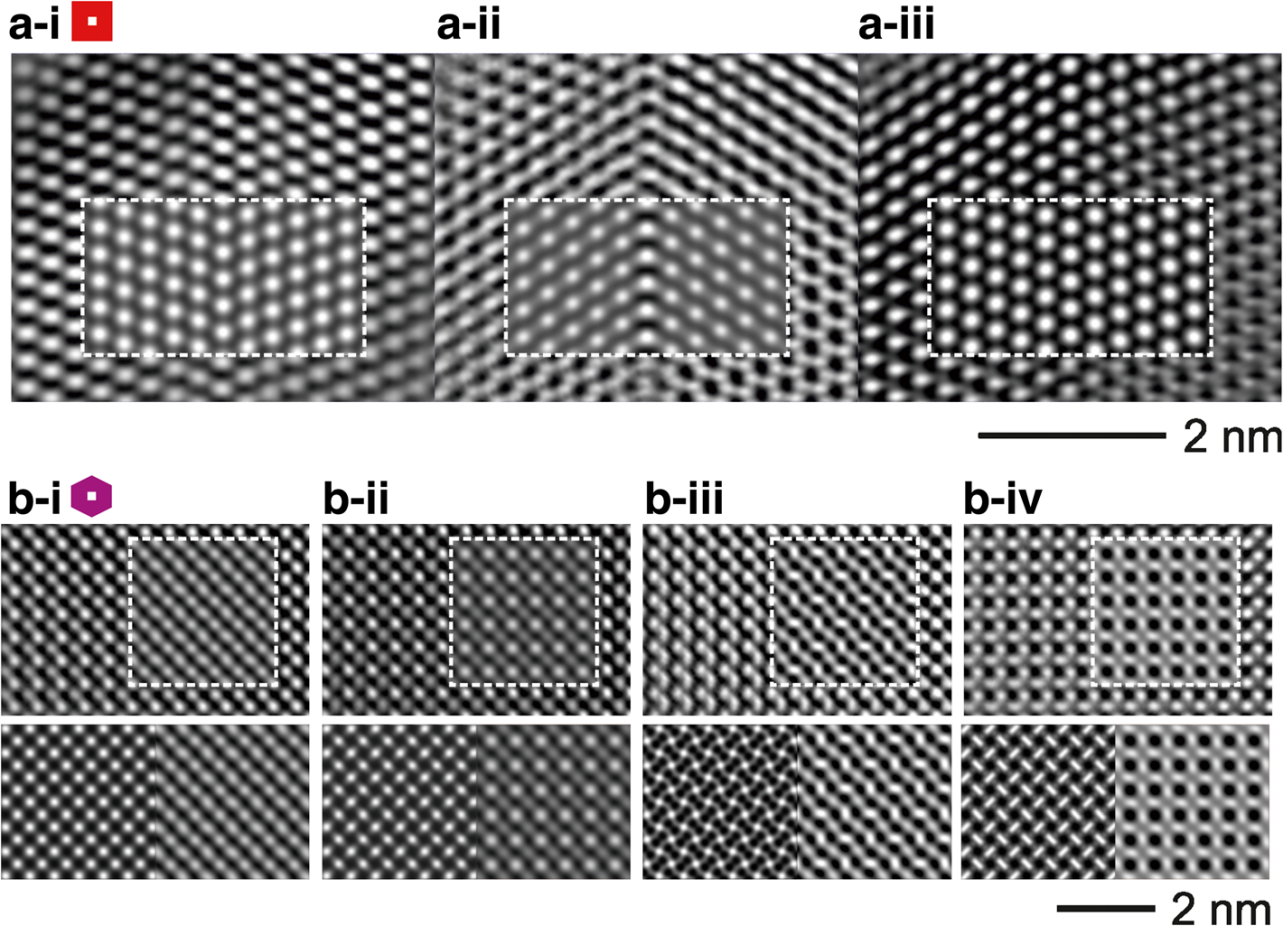
High resolution defocus series of the twin defect introduced in Fig. 5 for the edge directions **a** [010]^r^ and **b** [001]^r^ with inserted simulations. For **b** bottom panels: simulations of (left) rutile-type vs. (right) the defect model with corresponding parameter settings, respectively. The details of respective parameter setting (i, ii, iii, (iv)) are given in the text

The selected directions for the analyses are including the CTB in cross-section (i.e. [010]^r^ and [111]^r^) and indirect plane view (i.e. [001]^r^). High resolution contrasts were recorded for the marked directions and evaluated with simulations based on the modified separation model of step 3, cf. Fig. 5b: An excellent agreement between the respective calculation and experimental data-couples was achievable by identifying the parameter settings, i.e. objective lens defocus (Δf) and specimen thickness (t) for-i) [010]^r^: Δf = 0 nm - t = 4.73 nm, −ii) [111]^r^: Δf = − 52 nm - t = 4.45 nm and -iii) [001]^r^: Δf = 0 nm - t = 5.73 nm. All depicted high resolution contrasts exhibit distinct features, i.e. a clear edge-on view on the CTB (Fig. 5b-i), a strong contrast deviation of adjacent twin domains stemming from an oblique view to the CTB (Fig. 5b-ii), and a superposition contrast (Fig. 5b-iii). The perpendicular main directions [010]^r^ and [001]^r^ were selected for additional defocus series, as presented in Fig. 6. The series in Fig. 6a demonstrates the contrast change for the CTB with a specimen thickness of 4.73 nm and Δf of –i) 0 nm, −ii),-51 nm and -iii) -58 nm (close to the Scherzer defocus), respectively. The inserted simulations are in each case in convincing agreement to the experimental data, validating the quality of the defect model. Note, visible contrast deviations result from a strong thickness gradient propagating from bottom to top of each panel. The defocus series of the superposition structure along [001]^r^ (see Fig. 6) is of particular interest: At first glance the experimental data appear to be a conventional single domain contrast with no direct evidence of twinning influence. However, solely the defect model provides an experiment/calculation accordance by using the parameter settings Δf for b-i) 0 nm, b-ii) -5 nm, b-iii) -60 nm and b-iv) -95 nm with a common specimen thickness of 5.73 nm. The deliberate comparison of the single domain SnO_2_ rutile-type with the defect model simulations further evidences superposition twinning as origin for the high-resolution contrast, cf. the series of square panel comparisons in Fig. 6b. Note, other parameter settings for the rutile-type model exhibit even larger deviations to the presented experimental data.

### Tilting experiments II – reciprocal space

**Fig. 7:**
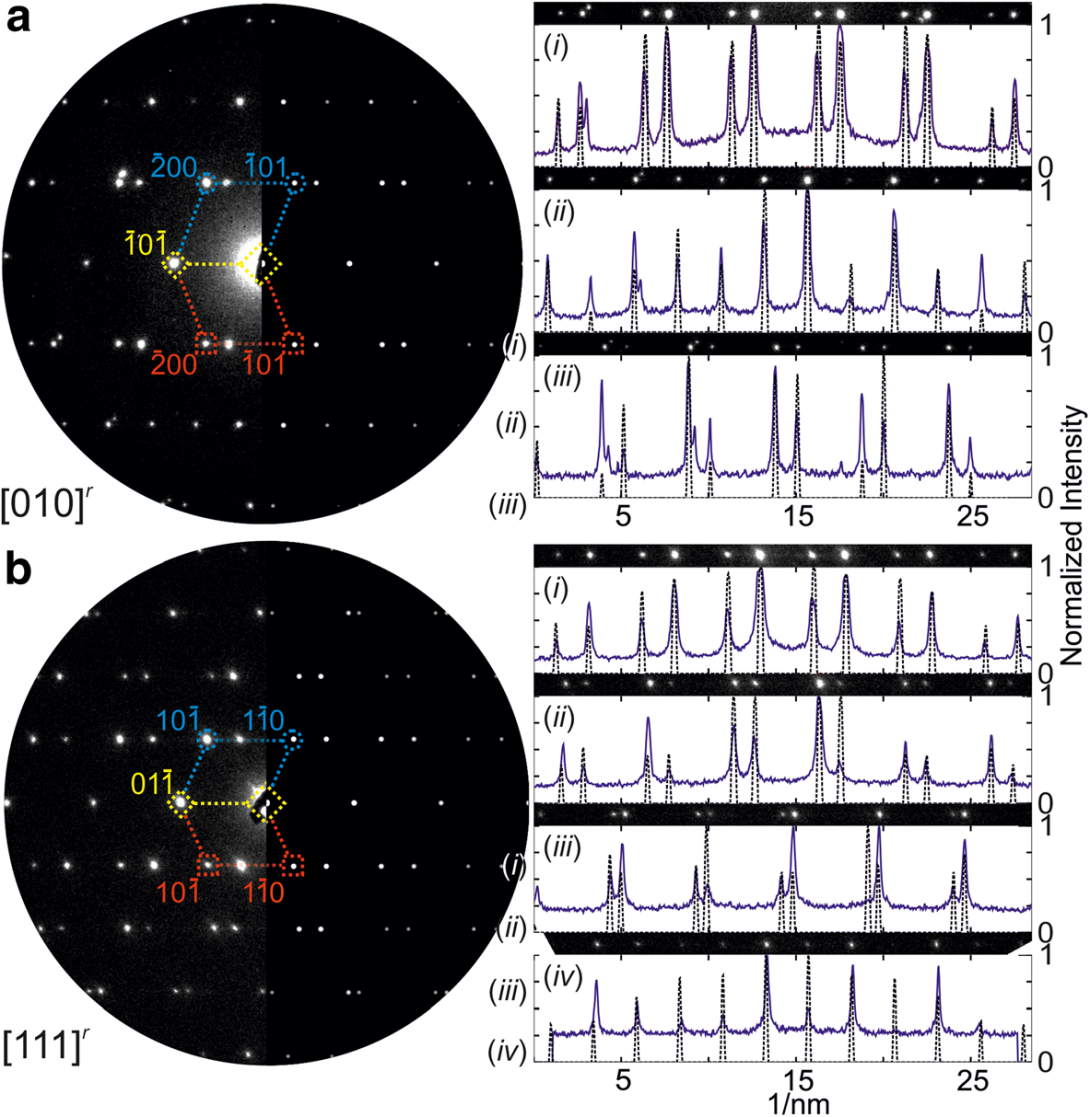
Experimental vs. simulated precession electron diffraction pattern data along **a** [010]^r^ and **b** [111]^r^. See text for details

Precession electron diffraction patterns of the CTB are compared with simulations based on the SPS in a plane view, cf. Fig. 7. Both, simulation and experimental ED pattern are merged for the respective direction (i.e., [010]^r^ in Fig. 7a and [111]^r^ in Fig. 7b) in one common representation, showing a good coinciding match of the intensity. An additional optical support is provided by graphical marks in the ED patterns: The (yellow) diamonds mark common reflections from both domains, while the (blue) circles and (red) squares emphasize the contributions from respective twin domains. Although a first considerable agreement of componential and experimental data can be imputed, a more substantial and critical assessment is achieved by a quantifying examination of the reflection rows, see the roman enumeration and intensity plots of Fig. 7. Straight (blue) lines represent the experimental profile measurements and the dashed (black) lines correlated the simulated equivalents. The high coincidence of associated reflection position validates the accuracy of the supercell approach with respect to geometrical (i.e. lattice) aspects. Considering the intensity distribution of the reflections, a deviation must be consternated. Such a difference is explainable by taking ratio of twin domain volumes into account. The simulation is calculated using the kinematic model and the same occupancy factor for all atoms in the SPS (i.e. one). As consequence, the twin domain ratio is 1:1. The experimental observation of such an idealized case appears to be highly improbable, in particular, considering the TEM preparation and aperture size (illuminated area: 100 nm) in ED mode for this study. Note, the variation of the occupancy factor and/or using the dynamic scattering model can lead to an improved matching between experimental and simulated intensities, see examples elsewhere (Hrkac et al. 2013). Additionally, appearing artifact reflections in the experimental PED pattern stem most probably from other grains.

**Fig. 8:**
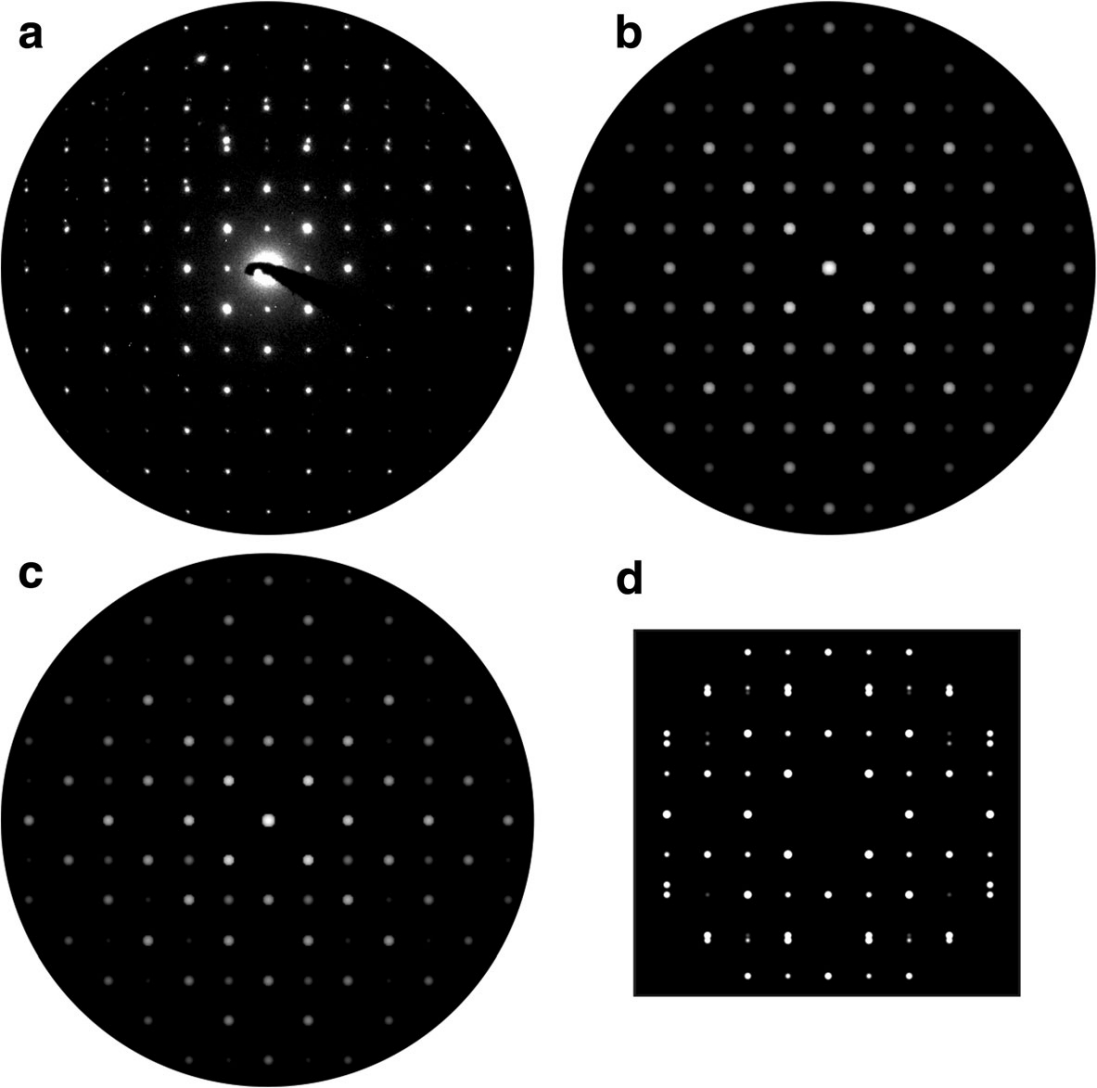
Superposition views of the coherent twin boundary along [001]^r^: **a** Experimental PED pattern along [001]^r^ vs. simulated ED pattern **b** based on a defect free rutile-type SnO_2_ model and **c** the SPS defect model. **b** and **c** based on the kinematic model. **d** Simulated PED including higher order Laue zones

A PED pattern was recorded along the [001]^r^ direction exhibiting the absence of a direct twinning indication, as presented in Fig. 8a. Three Bragg reflection types can be identified within the experimental data: The first type, fundamental Bragg reflections, stems from the *P*4_2_/*mnm* rutile-type, the second type, dynamical scattering reflections, are generated by the rather high specimen thickness (neglected for following simulations and discussions) and a third group, which exhibits with an asymmetric intensity distribution on the top hemisphere of the PED pattern. Note, the latter type exclusively appeared in PED mode, the corresponding selected area ED pattern was free of such feature. Kinematic simulations from both ideal rutile-type (Fig. 8b) and defect (Fig. 8c) model recreate the fundamental reflections with identical intensity ratios, consequently fail a clear assignment of the experimental data. An unambiguous identification of the reciprocal data was enabled by the corresponding real structure study illustrated in Fig. 6b. The third type reflections, at first glance, may be interpreted as debris reflections, originating from other grains accidently illuminated during the PED procedure. In some instances, randomly appearing reflections can be detected and explained in this manner. However, the third type reflections display a systematic characteristic, which matches, partially, a higher-order-Laue-zone-(HOLZ) including simulation of the defect model, cf. Fig. 8d. As two twinned components are involved in the formation of this PED pattern, HOLZ or also intersecting relrods from the additional component may cause the (asymmetric) presence of this feature. A further factor within the experimental data is the influence of a potential twin domain ratio deviating from unity. A minor domain will influence HOLZ and relrod formation and may also cause the asymmetry. Note, in other single domain data, experimentally and simulated patterns, no evidence of those third type reflections was found.

**Fig. 9:**
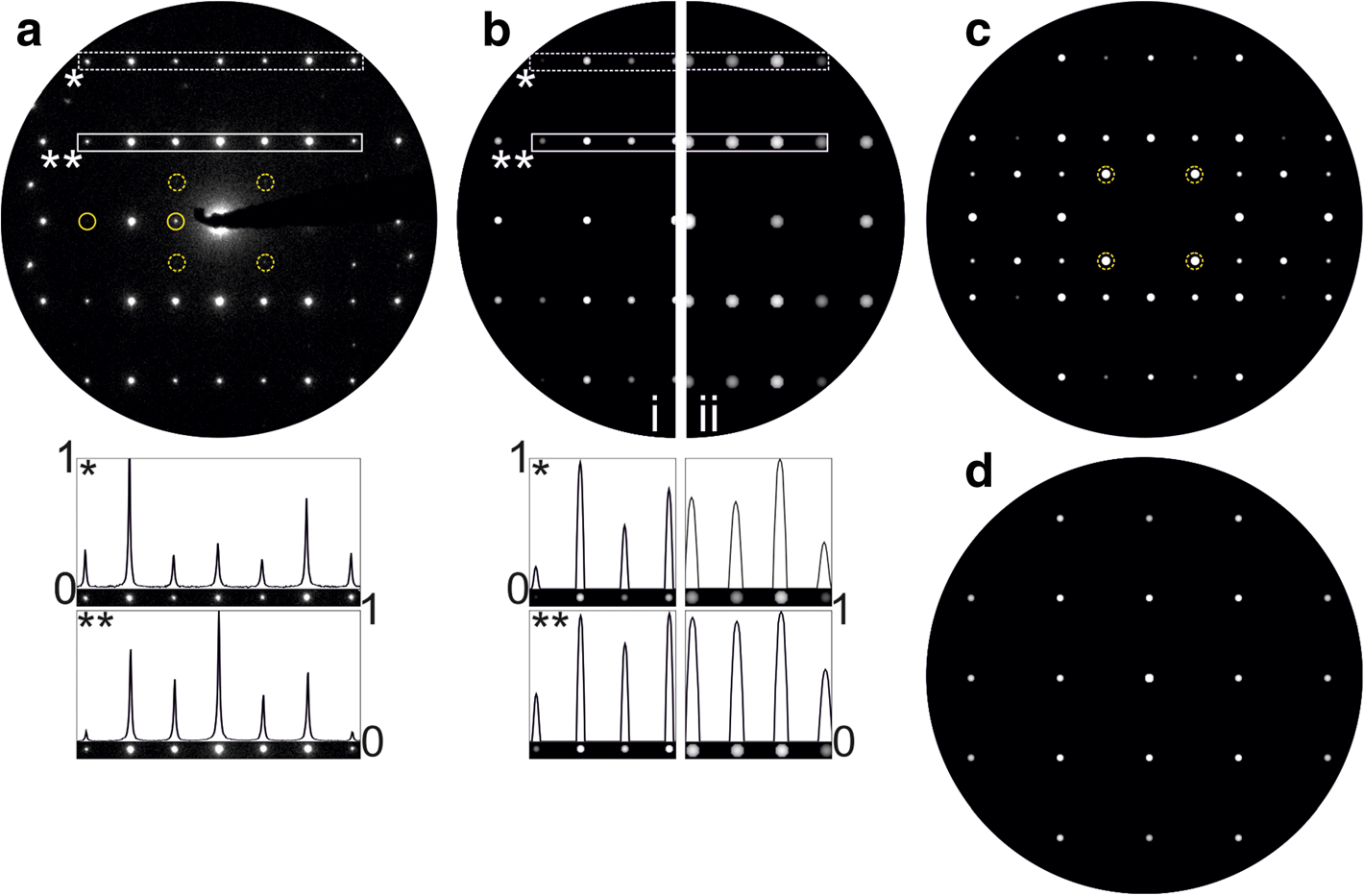
Electron diffraction study: **a** Experimental PED along [$\overline{3}$ 01]^t^, **b** kinematic simulations along: -i) [$\overline{3}$ 01]^t^ (defect model) and -ii) [101]^r^ (rutile-type model). Normalized intensity plots are adjacent to the marked areas, respectively. **c** PED simulation along [$\overline{3}$ 01]^t^ (defect model). **d** Kinematic simulation along [100]^t^ (defect model). The circular marked intensities are additional reflections with respect to the kinematic and defect-free rutile type model. See text for details

An additional example for superposition observation of the CTB was detected for the [101]^r^ direction, cf. PED pattern in Fig. 9a. The precession mode minimized the effects of dynamical scattering, allowing a detection of intensity variations within the Bragg reflection rows, cf. normalized intensity plots. The kinematic calculations from defect (ED along [$\overline{3}$ 01]^t^; b-i) and rutile-type model (ED along [101]^r^, Fig. 9b-ii) contain identical positions of the corresponding Bragg reflections and show a mutual good agreement to the experimentally observed reflections. An in-depth analysis of the patterns emphasizes a deviation in the intensity ratios and favors the defect structure as more appropriate interpretation of the experimental data, see normalized intensity plots. However, based only on these features a clear assignment of the experimental data to one of the structure candidates is impeded, due to the incomplete suppression of dynamical scattering. The most apparent evidence for the latter is the presence of the {101} reflections as marked with solid (yellow) circles.

The experimental PED pattern offers, despite the expected zero order (ZO)LZ reflections, most probably Bragg reflections stemming from HOLZ, i.e., the faint reflections marked with dashed circles (Fig. 9a). These reflections appeared exclusively in precession mode and are located on commensurable (h/2, k/2, l/2) lattice positions. A PED simulation based on the defect model reproduces these reflections, cf. marks in the Fig. 9c, and thus verifies HOLZ as potential origin. All HOLZ reflections possess comparably high intensities in the calculated patterns, differing to the general observation in the experimental pattern. As mentioned above, the discrepancy may result from a non 1:1 twin domain ratio and further dynamical scattering influences. It must be noted, that conventional ED simulation with sample thicknesses > 80 nm also shows these commensurable reflections. Another feature of the defect structure is emphasized with the diffraction study: While the directions [101]^r^ and [011]^r^ are completely symmetry equivalent in the rutile-type structure, the corresponding directions (i.e. [$\overline{3}$ 01]^t^ and [100]^t^, respectively) exhibit clear variation in the intensity ratios of the kinematic Bragg reflections, see Fig. 9c and d.

## Conclusion

In this study, a complete TEM characterization was discussed for one of the major defects, i.e. coherent twin boundary at (101)^r^, creating interconnected FTS-SnO_2_ 3D networks. The analytical process was enabled by deducing a set of defect models under the principle of a supercell approach. For the latter, exclusively crystallographic transformation is applied to the ideal rutile-type structure to develop a periodic structure with the observed defect plane incorporated. Comparison of calculated and experimental data exhibited excellent agreement for edge-on ([010]^r^, [111]^r^) and superposition ([001]^r^, [101]^r^) views of the defect structure. In particular, the identification of the pseudo single domain contrasts along the [001]^r^ direction was a clear evidence of the model validity. The generation of three-dimensional information from the two-dimensional diffraction pattern, was exclusively enabled by the defect model, and as consequence, quantitative statements of the atomic configuration at the twin interface were achieved.

## Abbreviations

3D: Three-dimensional
CTB: Coherent twin boundary
ED: Electron diffraction
EDS: Energy-dispersive X-ray spectroscopy
FFT: Fast Fourier transform
FTS: Flame transport synthesis
HOLZ: Higher order Laue zones
HRTEM: High resolution transmission electron microscopy
PED: Precession electron diffraction
Q1D: Quasi one-dimensional
SEM: Scanning electron microscopy
SnO_2_: Tin dioxide
SPS: Superposition structure
TEM: Transmission electron microscopy
